# Interspecific chloroplast genome sequence diversity and genomic resources in *Diospyros*

**DOI:** 10.1186/s12870-018-1421-3

**Published:** 2018-09-26

**Authors:** Wenqing Li, Yanlei Liu, Yong Yang, Xiaoman Xie, Yizeng Lu, Zhirong Yang, Xiaobai Jin, Wenpan Dong, Zhili Suo

**Affiliations:** 1 Shandong Provincial Center of Forest Tree Germplasm Resources, Jinan, China; 2 State Key Laboratory of Systematic and Evolutionary Botany, Institute of Botany, Chinese Academy of Sciences, Beijing, China; 3 University of Chinese Academy of Sciences, Beijing, China; 4 College of Horticulture, Northwest A&F University, Yangling, China; 5 Beijing Botanical Garden, Chinese Academy of Sciences, Institute of Botany, Beijing, China; 6 Peking-Tsinghua Center for Life Sciences, Academy for Advanced Interdisciplinary Studies, Peking University, Beijing, China

**Keywords:** *Diospyros*, Chloroplast genome, Hyper-variable region, Genetic diversity

## Abstract

**Background:**

Fruits of persimmon plants are traditional healthy food in China, Korea and Japan. However, due to the shortage of morphological and DNA markers, the development of persimmon industry has been heavily inhibited.

**Results:**

Chloroplast genomes of *Diospyros cathayensis*, *D. virginiana*, *D. rhombifolia* and *D. deyangensis* were newly sequenced. Comparative analyses of ten chloroplast genomes including six previously published chloroplast genomes of *Diospyros* provided new insights into the genome sequence diversity and genomic resources of the genus. Eight hyper-variable regions, *trnH-psbA*, *rps16-trnQ*, *rpoB-trnC*, *rps4-trnT-trnL*, *ndhF*, *ndhF-rpl32-trnL*, *ycf1*a, and *ycf1*b, were discovered and can be used as chloroplast DNA markers at/above species levels. The complete chloroplast genome sequences provided the best resolution at inter-specific level in comparison with different chloroplast DNA sequence datasets.

**Conclusion:**

*Diospyros oleifera*, *D. deyangensis*, *D. virginiana*, *D. glaucifolia*, *D. lotus* and *D. jinzaoshi* are important wild species closely related to the cultivated persimmon *D. kaki.* The hyper-variable regions can be used as DNA markers for global genetic diversity detection of *Diospyros*. Deeper study on these taxa would be helpful for elucidating the origin of *D. kaki.*

**Electronic supplementary material:**

The online version of this article (10.1186/s12870-018-1421-3) contains supplementary material, which is available to authorized users.

## Background

*Diospyros* is the largest genus in the family Ebenaceae, including more than 400 species all over the world, with a wide distribution and cultivation from tropical to temperate zones. Their fruits have been traditional healthy food source in China, Korea and Japan. Ebony trees are valued for their hard, heavy and dark timber. In addition, the bark, leaves, wood, fruits and seeds of several species are the main sources of medicines. *Diospyros kaki* is an important economic tree crop and widely cultivated species of the genus [1, 2].

In China, cultivation of persimmon plants (*Diospyros kaki*) for edible fruits and Chinese medicine is commonly one of the best choices for regional development of characteristic fruit tree industry, with different regions having different cultivars. At present, there are about 1000 persimmon cultivars in China. Most of the extant persimmon cultivars are elite plants from natural seedlings or bud mutation. Among these cultivars, a majority is of the pollination- constant and astringent (PCA) type, pollination-constant and non-astringent (PCNA) type of premature persimmon and disease-resistant cultivars are rarer and are highly desirable. In general, closely related wild species possess elite genetic background for qualitative improvement and high-yield breeding of the persimmon crops [1, 2].

Breeding of woody *Diospyros* plants is time-consuming, commonly around ten years being necessary for evaluating the performance of a hybrid plant. Due to the difficulty of identifying the *Diospyros* germplasm resource diversities, directive breeding through artificial hybridization is severely inhibited. Therefore, accumulation of genetic information for revealing the genetic diversity of *Diospyros* plants is in urgent need.

The germplasm resources of *Diospyros* plants have been documented in aspects of the use and phenotypic characteristics, such as fruit morphology, fruit color, fruit quality and floral characteristics. *Diospyros* plants have four ploidy levels (2n = 2× = 30; 2n = 4× = 60; 2n = 6× = 90; 2n = 9× = 135) according to their chromosome number [1–7]. However, due to the shortage and limited sensitivity of phenotypic and DNA markers, study on genetic differentiation detection techniques for the huge amount of *Diospyros* plants worldwide is still a global challenge [8–12].

In *Diospyros*, molecular resources have been developed in recent years for species identification. Some chloroplast genome markers (such as *rbcL*, *matK*, *trnH-psbA*) and nuclear DNA markers (e.g., internal transcribed spacer of ribosomal DNA, ITS) were used to discriminate plants in *Diospyros*. However, these markers are low in variability, or have limited resolution and cannot reach the goals [3, 13–18]. Development of more effective DNA barcodes is also important for *Diospyros* plants.

The angiosperm chloroplast genomes are uniparentally inherited with relatively stable structure [16, 17]. Accordingly, it is considered to be an informative and valuable resource for plant phylogenetic analyses at family/genus/species levels [16, 19–24]. In the past decades, the chloroplast genomes have been proven to be more powerful in revealing phylogeny of plants and resolving previously ambiguous taxonomic and phylogenetic issues [16–28].

In this study, we report newly sequenced complete chloroplast genomes of *D. cathayensis*, *D. deyangensis*, *D. rhombifolia* and *D. virginiana*. The aims of our study are: (1) to evaluate the variation in *Diospyros*; and (2) to develop new and efficient cpDNA markers for species identification in *Diospyros*.

## Methods

### Sampling and DNA extraction

The fresh leaves of the *Diospyros* taxa were collected in spring 2016 from the Beijing Botanical Garden of Chinese Academy of Sciences and the National Field Genbank for Persimmons of College of Horticulture, Northwest A&F University, Yangling, Shaanxi, China and were dried immediately using silica gels for DNA extraction. Total genomic DNAs were extracted following the procedure of Plant Genomic DNA Kit (DP305) from Tiangen Biotech (Beijing) Co., Ltd., China.

### Chloroplast genome sequencing, assembling and annotation

DNA was sheared to fragments of 400–600 bp with an ultrasonic disruptor. An Illumina paired-end library was constructed with the NEBNext® Ultra™ DNA Library Prep Kit according to the manufacturer’s protocol. Paired-end sequencing (2 × 150 bp) was conducted on an Illumina HiSeq 4000 platform. The high-throughput sequencing data were qualitatively assessed and assembled using SPAdes 3.10.1 [29]. Using the chloroplast genome sequence of *Diospyros kaki* (GenBank accession No. KT223565) as a reference sequence, we selected chloroplast genome contigs using the BLAST method. The contigs of the chloroplast genomes were assembled using Sequencher (v5.4) with default parameters. Ambiguous nucleotides or gaps and the four junction regions between the IRs and SSC/LSC in the chloroplast genome sequences were further confirmed by PCR amplification and Sanger sequencing with specific primers or the universal primers [20]. After that, all reads were mapped to the spliced chloroplast genome sequence using Geneious 8.1 [30] to avoid assembly errors. Chloroplast genome annotation was performed using the Dual Organellar Genome Annotator (DOGMA) [31]. The chloroplast genome map was drawn using Genome Vx software [32].

### Analysis of tandem repeats and single sequence repeats in chloroplast genomes

MISA (MIcroSAtellite; http://pgrc.ipk-gatersleben.de/misa) was used to detect simple sequence repeats (SSR) within the chloroplast genomes. Thresholds for a minimum number of repeat units were established as follows: > 10 for mono-nucleotide, > 5 for di-nucleotide, > 4 for tri-nucleotide, and > 3 for tetra-nucleotide, penta-nucleotide, or hexa-nucleotide SSR. Repeating sequences were scanned over the complete chloroplast DNA sequences, taxon by taxon, using the REPuter program.

### Sequence divergence analysis in chloroplast genomes

Four newly sequenced chloroplast genomes and six chloroplast genomes from GenBank were aligned using MAFFT [33] and manually adjusted using Se-Al 2.0 [34]. Variable and parsimony-informative base sites and genetic distance across the complete chloroplast genomes were calculated using MEGA 6.0 software [35]. Sliding window analysis was conducted to generate nucleotide diversity (Pi) of the chloroplast genomes using DnaSP (DNA Sequences Polymorphism version 5.10.01) software [36]. The step size was set to 200 bp, with a 600 bp window length.

### Chloroplast DNA barcoding analysis

Distance and tree based methods were used to evaluate discriminatory power of detected hyper variable regions and core DNA barcodes *rbcL* and *matK*. The function nearNeighbour of SPIDER was used for Distance method barcoding analyses [37]. Neighbor-Joining (NJ) trees were constructed by each hyper-variable marker and the different marker combinations using MEGA 6.0 based on a K2P distance model [35]. Relative support for the clades of the NJ tree was assessed via 1000 bootstrap replicates.

### Phylogenetic analysis based on chloroplast genome sequence data

Phylogenetic trees were constructed by Maximum Parsimony (MP), Maximum Likelihood (ML) and Bayesian Inference (BI) using the entire chloroplast genome.

MP analyses were performed using PAUP v4b10 [38] described in Dong et al. [24]. The ML analyses were conducted using RAxML 8.0 [39]. For ML analyses, the best-fit model, General Time Reversible, with gamma distribution (GTR + G) was used as suggested with 1000 bootstrap replicates. BI method was conducted with MrBayes v3.2 [40]. The Markov Chain Monte Carlo (MCMC) analysis was run for 2 × 5,000,000 generations with trees sampled every 1000 generations, with the first 25% discarded as burn-in. The remaining trees were used to build a 50% majority-rule consensus tree. The stationarity was regarded to be reached when the average standard deviation of split frequencies remained below 0.01.

## Results

### Chloroplast genome characterization

Previous experiments indicated that chloroplast genome sequences are identical among individual plants of the same species. Therefore, we sequenced four taxa of *Diospyros* (*D. cathayensis*, *D. virginiana*, *D. rhombifolia* and *D. deyangensis*) using an Illumina Hiseq 4000 system, 20,675,288 to 33,584,779 paired-end raw reads were obtained (average read length was 150 bp). After mapping the paired-end reads of each *Diospyros* taxon, 357,721 to 1,338,624 reads were extracted, yielding 340 × to 1271 × coverage (Table 1). The inverted repeat (IR) junction regions in assembled sequences were confirmed by using PCR-based sequencing. High quality chloroplast genome sequences were thus obtained and were used for downstream analyses. The four *Diospyros* chloroplast genome sequences were deposited in GenBank (accession numbers, MF288575- MF288578).

**Table 1:** Summary of the sequencing data for four *Diospyros* taxa

No.	Taxon	Raw data no.	Mapped read no.	Mapped to reference genome (%)	Chloroplast genome coverage (×)
1	*D. cathayensis*	25,027,276	533,606	2.13%	508
2	*D. deyangensis*	33,584,779	1,338,624	3.99%	1271
3	*D. rhombifolia*	20,675,288	915,369	4.43%	873
4	*D. virginiana*	30,770,623	357,721	1.16%	340

**Fig. 1:**
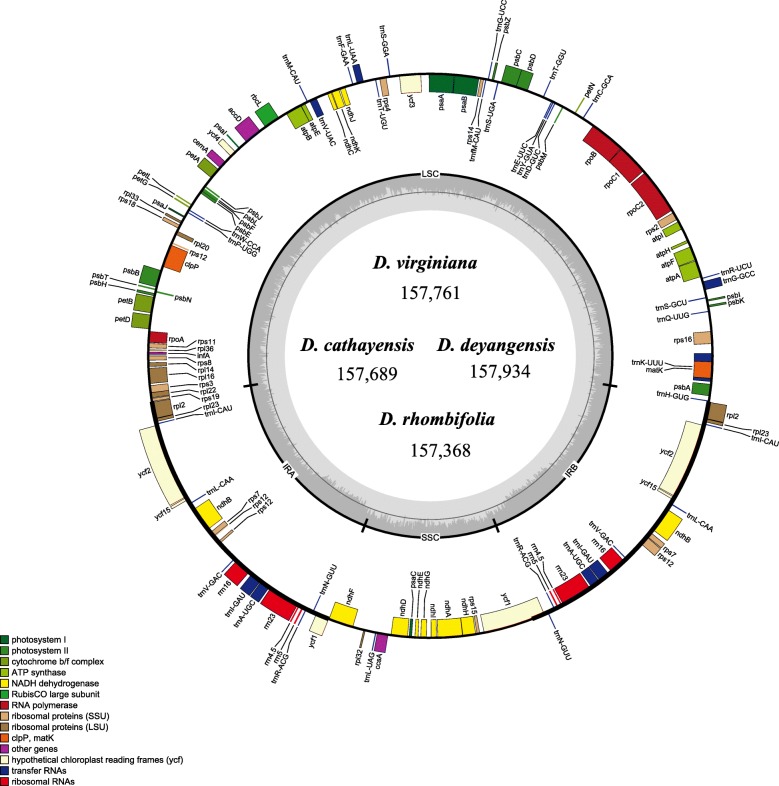
Gene map of *Diospyros* chloroplast genomes. The genes inside and outside of the circle are transcribed in clockwise and counterclockwise directions, respectively. Genes belonging to different functional groups are shown in different colors. The thick lines indicate the extent of the inverted repeats (IRa and IRb) that separate the genomes into small single-copy (SSC) and large single-copy (LSC) regions

The ten complete chloroplast genomes of *Diospyros* investigated in this study ranged from 157,300 base pairs (*D*. *jinzaoshi*) to 157,934 base pairs (*D*. *deyangensis*) in length. All the chloroplast genomes possessed the typical quadripartite structure of angiosperms, consisting of a pair of the inverted repeat region (IRs: 25,910–26,119 bp) separated by a large single-copy region (LSC: 86,948–87,246 bp) and a small single-copy region (SSC: 18,076–18,485 bp) (Fig. 1; Table 2). Each of the complete chloroplast genomes possesses 113 unique genes arranged in the same order, including 79 protein-coding, 30 tRNA, and 4 rRNA genes. The GC content of each chloroplast genome is identically 37.4% (Table 2). The genomic structure including gene number and gene order was highly conserved within *Diospyros* (Fig. 1; Table 2)*.*

**Table 2:** Complete chloroplast genome features of ten *Diospyros* taxa

Sample	GenBank accession No.	Genome size (bp)	LSC length (bp)	SSC length (bp)	IR length (bp)	Gene content	Protein coding genes	tRNA genes	rRNA genes	GC content (%)
*D. discolor*	KX426216	157,745	87,246	18,323	26,088	113	79	30	4	37.4
*D. cathayensis*	MF288576	157,689	87,176	18,349	26,082	113	79	30	4	37.4
*D. deyangensis*	MF288575	157,934	87,237	18,485	26,106	113	79	30	4	37.4
*D. glaucifolia*	KM504956	157,610	86,965	18,407	26,119	113	79	30	4	37.4
*D. jinzaoshi*	KM522848	157,300	87,010	18,076	26,107	113	79	30	4	37.4
*D. kaki*	KT223565	157,784	87,059	18,505	26,110	113	79	30	4	37.4
*D. lotus*	KM522849	157,597	86,948	18,411	26,119	113	79	30	4	37.4
*D. oleifera*	KM522850	157,760	87,034	18,532	26,097	113	79	30	4	37.4
*D. rhombifolia*	MF288578	157,368	87,223	18,325	25,910	113	79	30	4	37.4
*D. virginiana*	MF288577	157,761	87,089	18,444	26,114	113	79	30	4	37.4

### Comparative analysis of chloroplast genomes

**Fig. 2:**
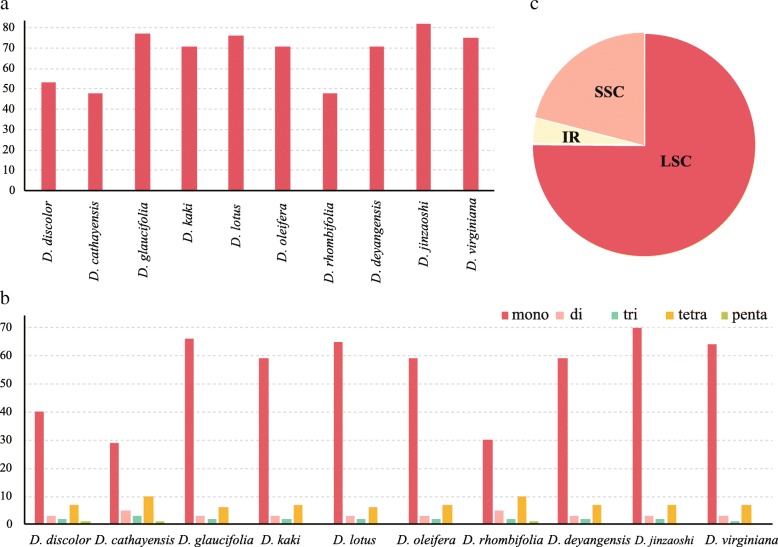
Comparison of simple sequence repeats among ten chloroplast genomes. **a**. Numbers of SSRs detected in ten *Diospyros* chloroplast genomes; **b**. Frequencies of identified SSRs in LSC, IR and SSC regions; **c**. Numbers of SSR types detected in ten *Diospyros* chloroplast genomes

The number of simple sequence repeats (SSRs) ranged from 48 (*D. cathayensis* or *D. rhombifolia*) to 82 (*D*. *jinzaoshi*) among the ten *Diospyros* taxa. The homopolymer repeat number with the highest variability, ranged from 29 (*D. cathayensis*) to 70 (*D*. *jinzaoshi*), while the number of di-nucleotide, tri-nucleotide or penta-nucleotide repeats had no significant difference among the ten *Diospyros* taxa (Fig. 2). The homopolymer repeats represented the major source of genetic diversity in *Diospyros*. In total, 505 SSRs were detected in LSC region, 141 in SSC region and 26 in IR region (Fig. 2).

**Fig. 3:**
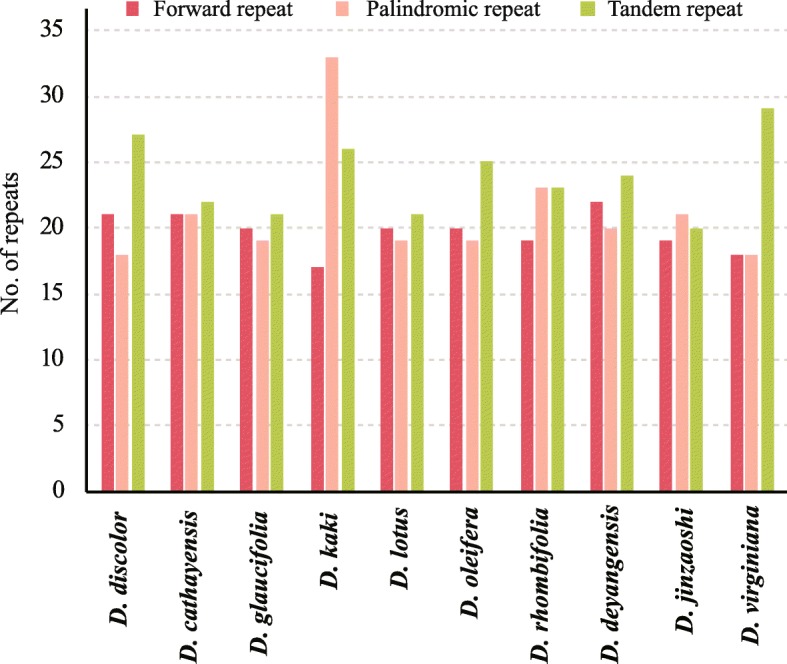
Numbers of repeats in comparison among ten chloroplast genomes

Four repeat types were detected in the chloroplast genomes using REPuter software. Forward repeat number ranged from 17 (*D. kaki*) to 22 (*D. deyangensis*), which were found to have no significant difference among the ten chloroplast genomes. Tandem repeat number ranged from 20 (*D*. *jinzaoshi*) to 29 (*D. virginiana*). Palindromic repeat number ranged from 18 (*D. discolor* or *D. virginiana*) to 33 (*D. kaki*). The highest value of palindromic repeat number which occurred in *D. kaki* is significantly different from that of other taxa. The highest tandem repeat number was observed in *D. virginiana* (Fig. 3).

The lowest value (31) of nucleotide substitution number was observed between *D. glaucifolia* and *D. lotus*, while the highest value (1493) of nucleotide substitution number occurred between *D. cathayensis* and *D. virginiana*, showing a wider range of variability according to the sequence alignment of the ten chloroplast genomes (Table 3).

**Table 3:** Numbers of nucleotide substitutions and sequence distance among the complete chloroplast genomes of ten *Diospyros* taxa

Taxon	*D. kaki*	*D. oleifera*	*D. deyangensis*	*D. jinzaoshi*	*D. glaucifolia*	*D. lotus*	*D. virginiana*	*D. cathayensis*	*D. rhombifolia*	*D. discolor*
*D. kaki*		0.0022	0.0022	0.0032	0.0046	0.0046	0.0068	0.0082	0.0081	0.0078
*D. oleifera*	346		0.0003	0.0029	0.0043	0.0043	0.0065	0.0079	0.0078	0.0074
*D. deyangensis*	348	42		0.0029	0.0043	0.0043	0.0065	0.0079	0.0077	0.0074
*D. jinzaoshi*	500	449	447		0.0043	0.0043	0.0066	0.0080	0.0079	0.0074
*D. glaucifolia*	723	673	676	673		0.0002	0.0074	0.0089	0.0088	0.0083
*D. lotus*	725	674	677	676	31		0.0074	0.0088	0.0088	0.0083
*D. virginiana*	1060	1017	1018	1029	1160	1161		0.0092	0.0091	0.0088
*D. cathayensis*	1280	1229	1228	1245	1381	1378	1493		0.0011	0.0070
*D. rhombifolia*	1263	1210	1207	1223	1366	1363	1419	168		0.0069
*D. discolor*	1211	1154	1151	1158	1295	1297	1366	1087	1081	

Notes: The lower triangle shows the number of nucleotide substitutions. The upper triangle indicates the number of sequence distance in complete chloroplast genomes

The sequence distance between *Diospyros* taxa ranged from 0.0002 to 0.0092. The smallest sequence distance occurred between *D. lotus* and *D. glaucifolia*, and the largest sequence distance occurred between *D. virginiana* and *D. cathayensis* (Table 3). LSC region was the most rapidly evolving region in the chloroplast genomes, while the IR region was the most slowly evolving region. The evolutionary rate of SSC region is moderate.

### Phylogenetic analysis

**Fig. 4:**
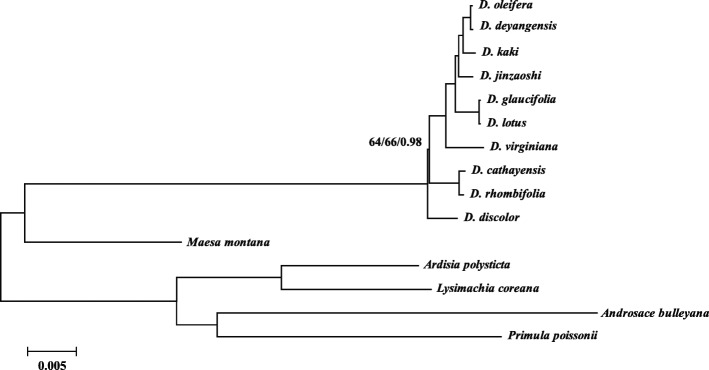
Phylogenetic relationships of ten *Diospyros* taxa inferred from Maximum Parsimony (MP), Bayesian Inference (BI), and Maximum Likelihood (ML) analyses of the complete chloroplast genomes. ML topology shown with ML bootstrap support value/Bayesian posterior probability/MP bootstrap support value given at each node. Nodes with 100 ML bootstrap support value/1.0 Bayesian posterior probability/100 MP bootstrap support value are not marked

Phylogenetic analyses indicated that all the ten taxa were clearly discriminated and seven clades were recognized among them (Fig. 4). The cultivars of *Diospyros* belonged to one clade of *D. kaki.* The DNA sequence data supported the isolated positions of *D. deyangensis* and *D. jinzaoshi* which were regarded previously as cultivars and have been suggested to rank to species level according to morphological and DNA characteristics by other recent researches [2–6]. The clade of *D. oleifera* and *D. deyangensis*, and the clade of *D. jinzaoshi* included the closest wild relatives of the cultivated persimmon plants of *D. kaki* (Fig. 4). Relatively speaking, the clade of *D. lotus* and *D. glaucifolia*, and the clade of *D. virginiana* have close genetic relationships with *D. kaki*. *D. lotus* and *D. virginiana* produce edible fruits, while *D. glaucifolia* is mainly used for timber production. *D. cathayensis* and *D. rhombifolia* that are genetically close with each other formed one clade. This is identical with their classification based on morphological characteristics. *D. discolor* is genetically the most distant taxon from the cultivated plants of *D. kaki* among the taxa investigated in this study.

### Chloroplast DNA marker development

**Fig. 5:**
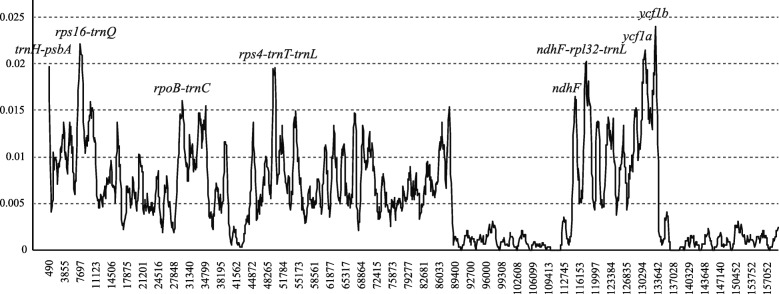
Sliding window analysis of the whole chloroplast genomes of nine *Diospyros* taxa (window length: 600 bp, step size: 200 bp). X-axis: position of the midpoint of a window; Y-axis: nucleotide diversity of each window

According to the chloroplast genome sequence alignment of the ten *Diospyros* taxa, eight hyper-variable regions, *trnH-psbA*, *rps16-trnQ*, *rpoB-trnC*, *rps4-trnT-trnL*, *ndhF*, *ndhF-rpl32-trnL*, *ycf1*a, and *ycf1*b were discovered (Fig. 5). These eight sequences could be used as DNA markers for classification and revealing the genetic divergence of the *Diospyros* taxa, with a high discrimination success ranging from 60 to 100% (Table 4), of which, the sequences of the three most rapidly evolving regions (i.e., *rps4-trnT-trnL*, *ndhF-rpl32-trnL*, and *ycf1*a) were able to discriminate all the taxa investigated in this study.

**Table 4:** Variability of nine hyper-variable markers and universal chloroplast DNA barcodes (*rbcL* and *matK*) in *Diospyros*

Marker	Length (bp)	Variable base sites		Informative base sites		Mean distance	Discrimination success (%) based on Distance method
		Number	Percentage (%)	Number	Percentage (%)		
*trnH-psbA*	1011	52	5.14	28	2.77	0.0178	80.0
*rps16-trnQ*	2184	122	5.59	65	2.98	0.0183	60.0
*rpoB-trnC*	885	46	5.20	22	2.49	0.0165	80.0
*rps4-trnT-trnL*	1469	72	4.90	39	2.65	0.0157	100.0
*ndhF*	1063	59	5.55	39	3.67	0.0166	80.0
*ndhF-rpl32-trnL*	2143	122	5.69	54	2.52	0.0182	100.0
*ycf1*a	2141	123	5.74	60	2.80	0.0181	100.0
*ycf1*b	1505	88	5.85	41	2.72	0.0177	80.0
Combined	12,401	684	5.52	348	2.81	0.0176	100.0
*rbcL*	1428	26	1.82	15	1.05	0.0057	40.0
*matK*	1512	46	3.04	24	1.59	0.0096	80.0

In those most rapidly evolving regions, 72, 122 and 123 variable base sites were detected, respectively, of which, 39, 54 and 60 informative base sites, made up 2.52–2.80% in each of the sequences. Comparatively, the commonly recommended DNA fragments (*rbcL* and *matK*) achieved only 40% and 80% of discrimination success respectively (Table 4, Additional file 1: Figure S1).

**Fig. 6:**
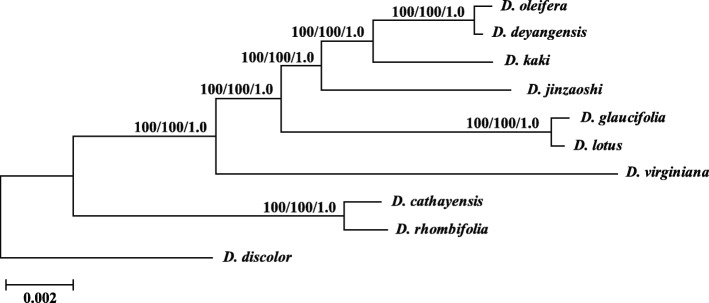
Phylogenetic relationships of ten *Diospyros* taxa inferred from Maximum Parsimony (MP), Bayesian Inference (BI), and Maximum Likelihood (ML) analyses using the chloroplast DNA sequences from eight hyper-variable regions

Similar results were obtained when different methods were used for phylogenetic tree reconstruction (Additional file 1: Figure S1). The combined sequence data from the eight hyper-variable cpDNA regions provided a well-resolved phylogenetic topology of the ten *Diospyros* taxa, similar to the topology obtained using complete chloroplast genome sequences, but with much lower experimental cost (Fig. 6). All the nodes in the phylogenetic tree received high bootstrap value (100%).

## Discussion

### Chloroplast genome variation and evolution

In this study, we sequenced four chloroplast genomes of *Diospyros* using Illumina Hiseq-4000 platforms and compared these genomes with the other six published chloroplast genomes downloaded from GenBank. The chloroplast genomes of *Diospyros* displayed the typical quadripartite structure of flowering plants, were conservative in gene order and gene content, in comparison with the most lineages of angiosperms. The chloroplast genomes ranged from 157,300 to 157,934 bp in length. IR expansion and contraction and the occurrence of larger indels (insertion/deletion) are considered to be the primary mechanisms affecting length variation of angiosperm chloroplast genomes. Only minor variations were detected at the SC/IR boundaries of the ten *Diospyros* chloroplast genomes. Occurrence of indels was the main factor effecting the variation of the length in *Diospyros* chloroplast genomes. Similar to previously published chloroplast genomes of the angiosperms, the *Diospyros* chloroplast genomes contained more AT content than GC content.

Simple sequence repeats (SSRs) are genetically variable molecular markers and are used widely in population genetics [41, 42], polymorphism investigations, and phylogenetic analyses [43]. Using MISA, the SSRs in the ten *Diospyros* chloroplast genomes were identified. The number of SSRs ranged from 48 to 82 in *Diospyros*, similar to those in *Lagerstroemia* [28]. SSRs with AT richness have been reported in other plants [44, 45]. Homopolymers are the most common SSRs in chloroplast genomes. Since the structure and organization of chloroplast genomes are conservative, SSR primers are transferable across species, genera, and even families. Information involving SSRs in this study will provide useful sources for estimating the genetic diversity and studying phylogenetic relationships among species and genera.

### Potential cpDNA barcodes

*Diospyros* is the largest genus in its family, including more than 400 species all over the world. For effective exploration, conservation, and domestication, accurately identified wild species would provide a clear genetic background of this genus. However, the taxonomic inventory of *Diospyros* still has a long way to go, because of the vast amount of species with extensive global distribution and the plasticity of the morphological characteristics. DNA barcoding has been largely used as a new biological tool to facilitate accurate species identification [46]. The two chloroplast DNA regions, *rbcL* and *matK*, are recommended as core universal DNA barcodes in plants. Therefore, genomic comparative researches of more complete chloroplast genome sequences have become necessary for developing variable DNA barcodes. These mutation “hotspot” regions could provide adequate genetic information for species identification, and can be used to develop novel DNA barcodes [19]. The eight potential mutational hotspots (*trnH-psbA*, *rps16-trnQ*, *rpoB-trnC*, *rps4-trnT-trnL*, *ndhF*, *ndhF-rpl32-trnL*, *ycf1*a, and *ycf1*b) identified in this study could be suitable barcodes for plant classification in *Diospyros.* In previous study, *ycf1* gene showed high divergence in chloroplast genome and was recommended as core DNA barcode for plants [22]*. Ycf1* gene has been more and more widely applied in plant phylogeny and DNA barcoding studies [47–51]*. TrnH-psbA, ndhF* and *rps16-trnQ* were popular candidates for phylogenetic studies [52, 53]*. Rps4-trnT-trnL* and *ndhF-rpl32-trnL* were newly identified in this study.

Recently, using the chloroplast genome as a super-barcode for plant species identification was discussed [49]. The analyses on chloroplast genome sequence divergence and phylogeny showed that the chloroplast genome may indeed be useful as a super-barcode for species identification of *Diospyros* (Table 3 and Fig. 4)*.* Further research is necessary to investigate whether these hyper-variable regions or complete chloroplast genome sequences could be used as reliable and effective DNA barcodes for species of *Diospyros*. The results obtained in this study have significant value for future studies on global genetic diversity assessment, phylogeny, and population genetics of *Diospyros*.

### Perspectives of persimmon research in future

It is important and fundamental to develop efficient identification methods and elucidate the genetic relationship of *Diospyros* taxa for planning breeding strategies, intensive management and conservation of *Diospyros* germplasm resources. The origin of *D. kaki* and its relationship to other *Diospyros* species have been also hot issues attracting scientists. Ng [54] suggested that *D. oleifera* was a parent of *D. kaki* based on morphological, geographical and cytological evidences. Yonemori et al. [55] suggested that *D. virginiana* and *D. kaki* had close relationship, as revealed by restriction fragment length polymorphism of chloroplast DNA. However, Choi et al. [9] considered the relationship between *D. virginiana* and *D. kaki* to be possibly phylogentically more remote than others.

The DNA evidences obtained in this study clearly support the previous taxonomic proposals that *D. deyangensis* and *D. jinzaoshi* should be raised to species rank based on morphological, molecular characteristics and chromosome numbers [4, 9–12]. In the cpDNA–based tree, the *D. kaki* lineage and the lineage including the dioecious *D. deyangensis* and the monoecious *D. oleifera* shared a common furcation. Interestingly, the chromosome number of *D. kaki* (2n = 6× = 90) is equal to the sum of the chromosome numbers of *D. deyangensis* (2n = 4× = 60) and *D. oleifera* (2n = 2× = 30)*.* This strongly implied that cultivated *D. kaki* might be resulted from a superposition event of genetic materials between *D. deyangensis* and *D. oleifera* through certain genetic mechanism during the evolutionary history of *Diospyros* plants. *D. oleifera* is naturally distributed south of the Changjiang River in China, has strong adaptability and resistance to wet condition, and has been used as rootstocks of *D. kaki* in southern China. Its fruits can also be used for extraction of persimmon paint. *D. deyangensis*, naturally distributed in Sichuan Province, China, produces red flowers and has fine hairs on the surface of the leaves, shoots, fruits, and some parts of the flowers [7].

*Diospyros rhombifolia* and *D. cathayensis* are genetically remote from *D. kaki*, being identical with the classification based on phenotypic characteristics*. D. rhombifolia* could not be used as rootstocks or inter-stocks because of its dwarfing effect [2]. *D. glaucifolia* and *D. lotus* were clustered together based on cpDNA sequences. They are very similar in phenotypic characteristics [2, 5, 6, 55]. *D. lotus* has made great contributions as rootstocks in persimmon production areas of northern China because of its highest cold resistance in the genus. The small fruits of *D. lotus* have been used as food and Chinese medicine with a long history. In southern China, the dioecious species *D. glaucifolia* is used to produce wood and could also be used as rootstocks*.* The species boundary between *D. lotus* and *D. glaucifolia* should be further studied by sampling more individual plants in future [2, 5, 6, 56].

Elucidation of the questions mentioned above would definitely improve our understanding on phylogeny, relationship, and the origin of the cultivated persimmons in *Diospyros*, and further accelerating directive breeding of *Diospyros* plants.

## Conclusion

Chloroplast DNA sequences can be used for classification of *Diospyros* plants at inter-specific level. The results obtained in this study implied that *D. oleifera*, *D. deyangensis*, *D. virginiana*, *D. glaucifolia*, *D. lotus* and *D*. *jinzaoshi* are important wild species closely related to the cultivated persimmon *D. kaki* and deeper studies on these taxa would be helpful for understanding the origin of *D. kaki.* Our results have significant value for global genetic diversity assessment, phylogeny, and population genetics in *Diospyros* in future.

## Additional file

Additional file 1: **Figure S1.** NJ trees of *Diospyros* taxa based on each of the ten chloroplast barcodes, showing the resolutions of each different locus for revealing the phylogeny. The figures above the lines are the bootstrap support values for the clades. (PDF 506 kb)

## Abbreviations

BI: Bayesian Inference
cpDNA: Chloroplast DNA
DnaSP: DNA Sequences Polymorphism
DOGMA: Dual Organellar Genome Annotator
GTR: General time reversible
Indel: Insertion/deletion
IR: Inverted repeat
ITS: Internal transcribed spacer of ribosomal DNA
LSC: Large single copy
MCMC: Markov Chain Monte Carlo
ML: Maximum Likelihood
MP: Maximum Parsimony
NJ: Neighbor-Joining
PCA type: Pollination-constant and astringent type
PCNA type: Pollination-constant and non-astringent type
Pi: Nucleotide diversity
rRNA: Ribosomal RNA
SSC: The small single copy
SSR: Simple sequence repeats
tRNA: Transfer RNA
