# Supplementary figures and images for: Interspecific chloroplast genome sequence diversity and genomic resources in Diospyros
Source: BMC Plant Biol. 2018 Sep 26;18:210. doi: 10.1186/s12870-018-1421-3 (PMC6158880; doi:10.1186/s12870-018-1421-3)

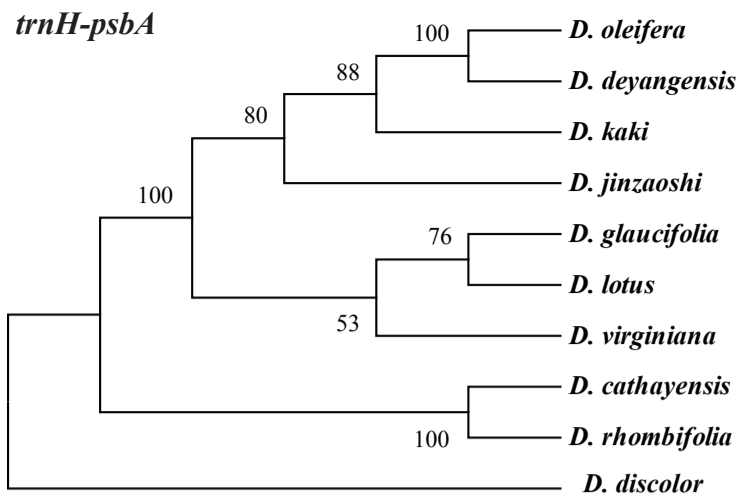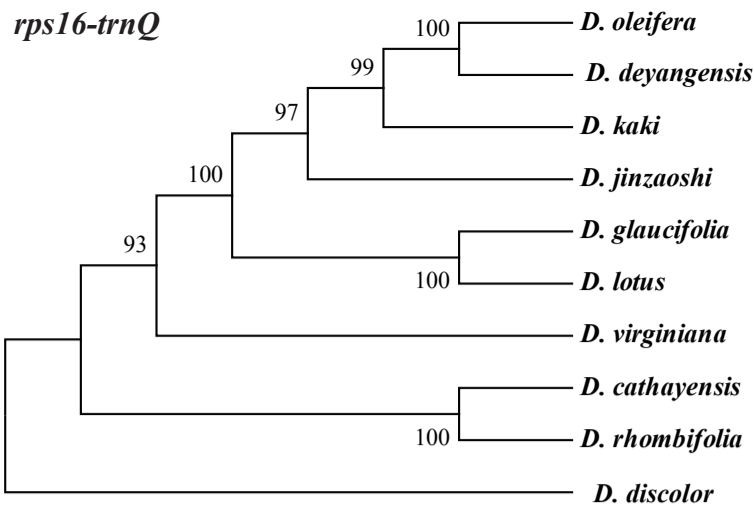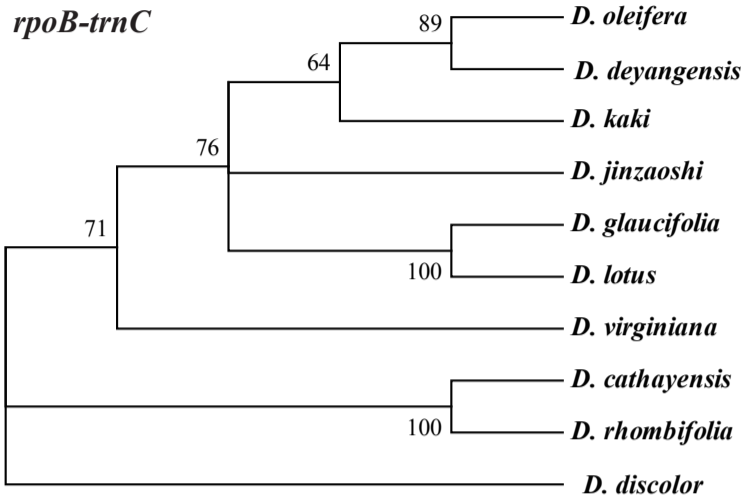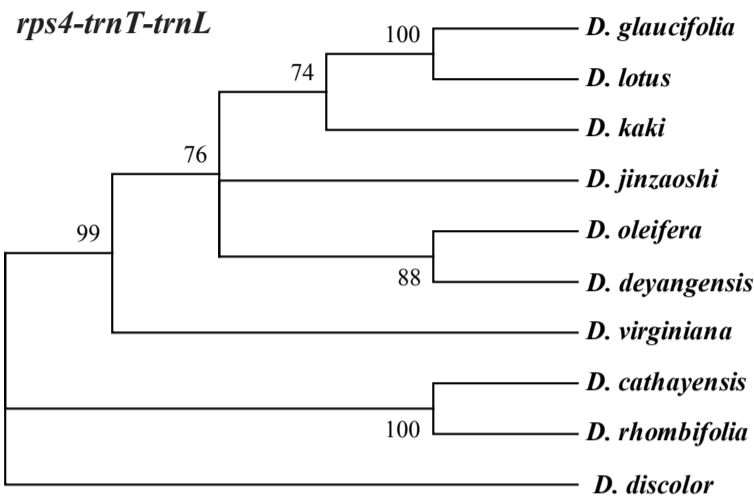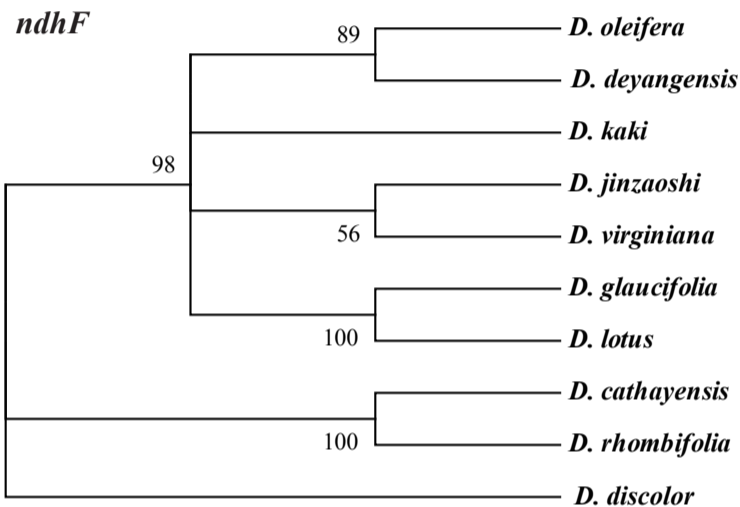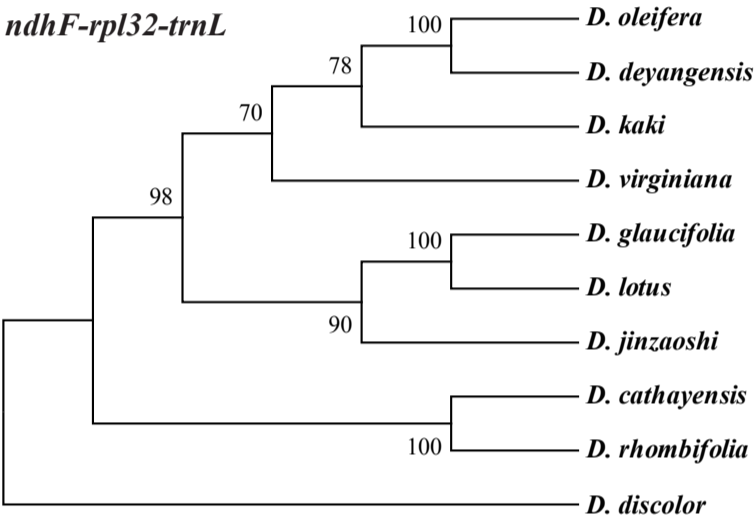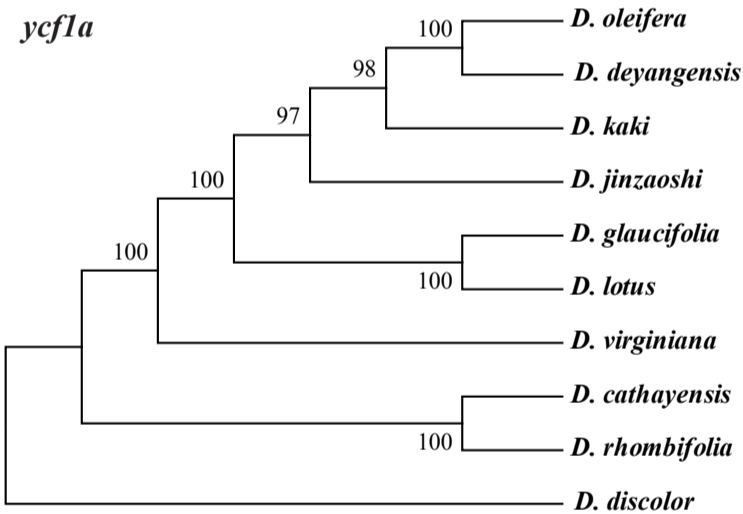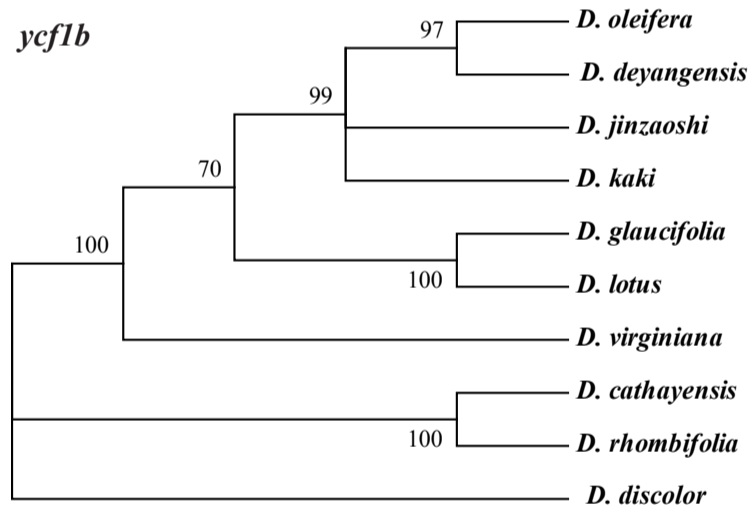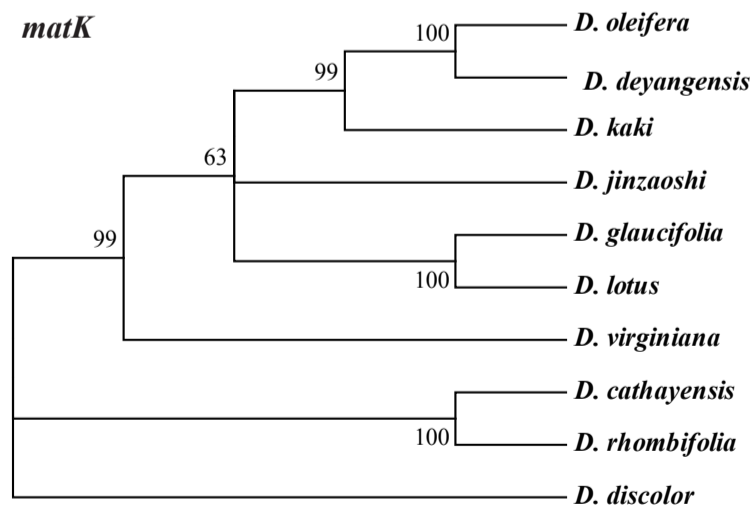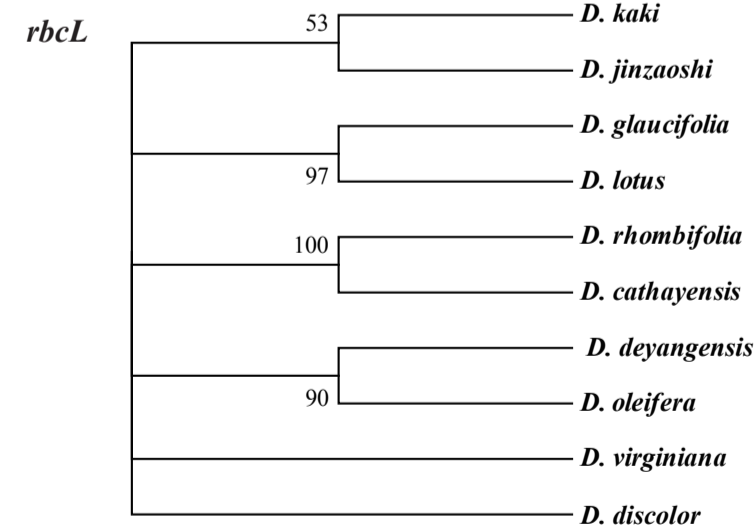

Supplement: Supplementary file 1 — Figure S1. NJ trees of Diospyros taxa based on each of the ten chloroplast barcodes, showing the resolutions of each different locus for revealing the phylogeny. The figures above the lines are the bootstrap support values for the clades. (PDF 506 kb) [file 12870_2018_1421_MOESM1_ESM.pdf]
